# On the Distinctive Hardness, Anti-Corrosion Properties and Mechanisms of Flame-Deposited Carbon Coating with a Hierarchical Structure in Contrast to a Graphene Layer via Chemical Vapor Deposition

**DOI:** 10.3390/nano12172944

**Published:** 2022-08-26

**Authors:** Wei Meng, Jinbin Zou, Xingyao Wang, Peng Zhang, Xusheng Du

**Affiliations:** Institute of Advanced Wear & Corrosion Resistant and Functional Materials, Jinan University, Guangzhou 510632, China

**Keywords:** graphene coatings, carbon, hardness, corrosion, scratch, grains and interfaces

## Abstract

Two carbonaceous (amorphous carbon and graphene) coatings were catalytically grown on bulk Ni plates. It was found that the flame-deposited carbon (FDC) layers exhibited a unique hierarchical structure with the formation of FDC/Ni nano-interlocking interface. The effect of the flame coating time on its corrosion protective efficiency (PE) was studied and compared with that of graphene coating produced via chemical vapor deposition. The FDC grown for 10 min exhibited a PE of 92.7%, which was much greater than that of the graphene coating (75.6%). The anti-corrosive mechanisms of both coatings were revealed and compared. For graphene coatings, the higher reaction temperature than that for FDC resulted in large grain boundaries inherent in the coating. Such boundaries were weak points and easily initiated grain boundary corrosion. In contrast, corrosion started at only certain local defects in FDC layers, whose unique interface structure likely promoted its PE as well. Moreover, after the coating process, the hardness of FDC-coated Ni remained almost unchanged, in contrast to that of graphene-coated samples (reduced by ~30%). This is suggested to be related to the crystal structure evolution of the Ni substrate caused by the heat treatment accompanying the coating process.

## 1. Introduction

Due to its excellent chemical inertness, high thermal stability, environmental friendliness, and cost effectiveness, carbon-based coating has been investigated as one type of the most important surface protection materials. Various carbon coatings have been developed so far, including the conventional diamond-like carbon [1,2,3] and graphite-like carbon [4,5,6] coatings. As a two-dimensional (2D) carbonaceous material composed of sp^2^-hybridized carbon atoms, graphene is impermeable to most small molecules and possesses exceptional chemical/thermal stability, which makes it a promising protective barrier for the underlying substrate. Graphene coatings have been demonstrated to be highly effective in protecting Cu and Cu/Ni alloy substrates from oxidation [7]. Research on the graphene-based anti-corrosive coatings has been attracting increasing attention [8,9,10,11,12,13,14,15,16,17,18,19,20,21,22,23]. For example, graphene/epoxy coatings were fabricated and displayed outstanding protection performance due to the maximized barrier effect through the well-controlled distribution of graphene in the coating [13]. In situ grown or transferred graphene coating exhibited effective protection performance of metal substrates in a harsh environment. With a graphene coating, a fivefold improvement in the corrosion resistance of nickel-plated stainless-steel plates could be achieved. This suggests their potential utilization as high-performance bipolar plates in the highly corrosive environment of a Polymer Electrolyte Membrane Fuel Cell [12]. For the tubular graphene-coated Ni wires, the corrosion protective efficiency (PE) was measured at 77.6% in 0.1 M HCl [15]. In addition, the graphene layer deposited on Ni by laser irradiation also displayed a PE of 68.9% in the same corrosive environment [11]. Meanwhile, although some advanced work has been done, more comprehensive research is worth conducting to reveal the corrosive mechanism of the graphene-coated samples.

Due to its time-saving, energy-conserving, and eco-friendly characteristics, and its convenience of operation, the flame synthesis of carbon materials has drawn growing attention. Carbonaceous nanomaterials in various forms, including 3D foam, 2D layer and 1D nanotube/nanofiber, have been deposited by this method on various substrates recently, including metals [24,25,26], inorganic oxides [27] and carbon fibers [28]. However, little information on the surface protection performance of the flame-deposited carbon (FDC) layers in comparison with its graphene counterpart is available at present, as well as on their anti-corrosion mechanisms. Additionally, the mechanical properties of the metallic substrate could be unexpectedly changed, as the carbon deposition process will apply heat treatment to it simultaneously. Since little research has been directed toward this aspect to the best of our knowledge, it is valuable to study the effect of the different carbon deposition techniques on the mechanical properties of the Ni substrate.

Herein, graphene coatings will be fabricated via CVD on bulk Ni substrate for surface protection, and FDC layers will be catalytically grown on the same catalytic metal substrate and carbon source as well. Their structure and properties will be investigated in comparison with each other. The FDC layers with unique hierarchical structure were found to be flame-deposited on Ni within a few minutes. The influence of the carbon deposition on the micro-hardness, the scratch, and corrosion resistance of bulk Ni substrate will be investigated based on the comprehensive characterization of the physico-chemical structure of both the FDC and graphene-coated samples. The corrosion mechanisms for both the FDC and graphene coating will be studied and proposed as well, including the role of FDC/Ni interlocking interface and the grain boundaries of graphene-coated Ni substrate.

## 2. Materials and Methods

Ni plates were polished with 3.5-micron diamond polishing paste and cleaned in an ultrasonic cleaner. Acetone, alcohol and water were used, respectively, to ultrasonically clean the Ni plates for 10 min in sequence, to ensure the complete removal of diamond abrasive particles. After that, the samples were dried in an oven at 60 °C for 30 min, followed by being cut into small plates with the dimensions of 10 mm × 10 mm. The carbon deposition was carried out by inserting the plates into the flame generated by the alcohol burner. They were treated in the flame zone with a temperature of 400–450 °C for 1 min, 3 min, 5 min, 10 min and 15 min, the respective specimens being labeled as C/Ni-1, C/Ni-3, C/Ni-5, C/Ni-10 and C/Ni-15. In order to characterize the flame-derived carbon coating in detail, the Ni substrate in the C/Ni-3, C/Ni-5 and C/Ni-10 specimens was etched off by HCl, and the obtained carbon layer was labeled as C-3, C-5 and C-10, respectively.

For the CVD deposition of the graphene coating, the polished Ni plates were placed into the tube furnace. After being treated for 10 min at 1000 °C in an ethanol/N_2_ atmosphere, the graphene-coated Ni sample was obtained and labeled as G/Ni-10. Similarly, a graphene layer was obtained by the removal of the Ni substrate with chemicals, and labeled as G-10.

Raman spectrum measurements were obtained under a 633 nm laser light (Invia Renishaw Raman, Horiba, Vignate Gieres, France) with the wavenumber range from 800 to 3000 cm^−1^. ZEISS ultra plus FESEM was used to detect the surface and structure of the carbon coatings. X-ray diffraction (XRD) was conducted on a Rigaku Ultima IV X-ray diffractometer (Rigaku, Nagano City, Japan). Transmission electron microscopy (TEM) was performed on a JEOL 2100F (JEOL, Beijing, China). A scratch test was conducted on ZKKH WS-2005 scratch tester together with a KOPPACE KP-200MRT microscope (KOPPACE, Shenzhen, China). The C/Ni-10 sample was scratched with a diamond indenter (120° core angle) under an increased normal load from 0 to 50 N, at a loading rate of 50 N min^−1^, and a scratching speed of 4 mm min^−1^. Optical photos of the immersion test were taken with a Leica DMI 3000 m inverted microscope. The hardness of bare Ni and coated samples had been measured with a Vickers hardness tester (HXD-1000TMSC/LCD), a load of 0.5 N (HV 0.05) was used to test the hardness of the samples according to the standard GB/T 4340.2-2012.

The electrochemical study of the corrosion behavior of the specimens was conducted in 0.1 M HCl. All electrochemical tests were carried out on a CHI 760E electrochemical workstation. The typical three-electrode configuration consists of a Pt foil as counter electrode, an Ag/AgCl reference electrode and specimen with 1 cm^2^ exposed area as work electrode. Potentiodynamic polarization curves were swept from −0.6 V to 0.4 V (vs. Ag/AgCl). EIS tests were performed at open circuit potential in a range of 10^5^–10^−2^ Hz, with amplitude of 5 mV.

## 3. Results and Discussion

### 3.1. Microstructure and Morphology of the Coating

**Figure 1:**
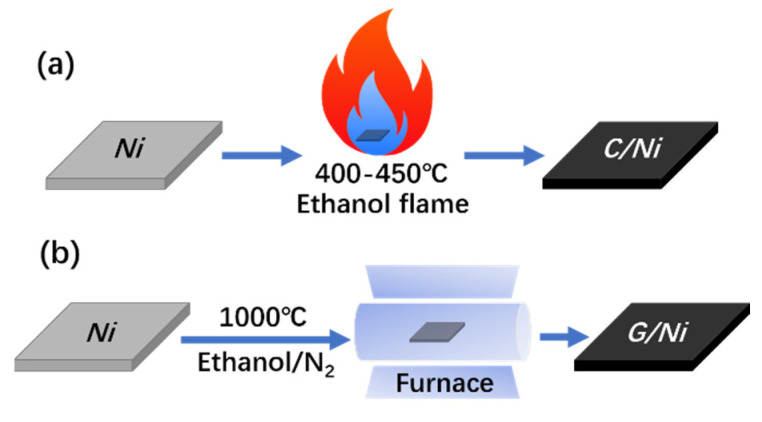
Schematic diagram of the coating process via: (**a**) flame deposition of carbon and (**b**) CVD of graphene coating.

A schematic diagram of the flame coating process is presented as Figure 1a. Generally, the inner flame zone provides a perfect environment, with active carbonaceous species derived from the incomplete combustion of the fuel and high temperature (400~450 °C) simultaneously. Polished Ni plates in the coating technique not only acted as the metal substrate but also as catalysts for the dehydrogenation of active carbonaceous species, which was followed with carbon dissolution and the segregation process for carbon deposition on their surface [29,30,31]. Bare Ni plates displayed a shiny silver gray color. In contrast, their surface turned increasingly darker with the flame treatment time. This phenomenon meant that a uniform and integrated carbon layer was successfully deposited onto the metal surface. For comparison, a graphene-coated Ni sample was also fabricated via a common CVD approach (Figure 1b). To keep the carbon source the same as that in the flame coating technique, ethanol was also utilized as the fuel in the CVD process.

**Figure 2:**
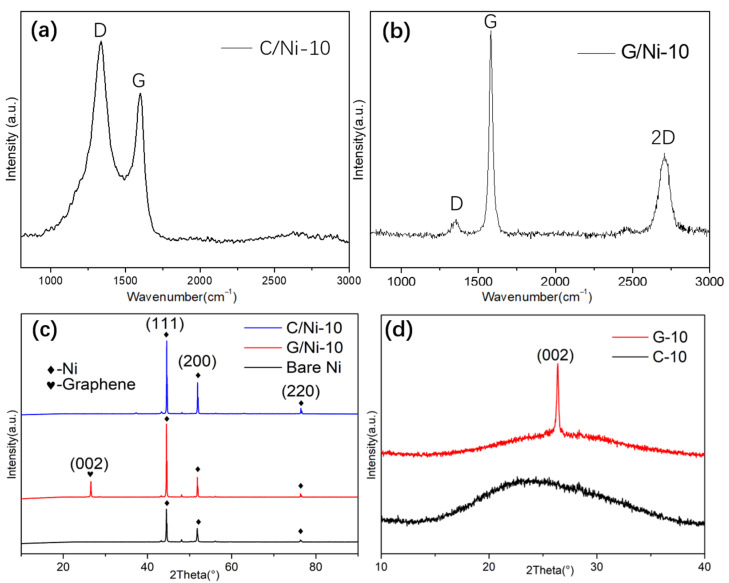
Raman spectra of C/Ni-10 (**a**) and G/Ni-10 (**b**), (where D band represents disordered carbon, G band represents graphitic carbon, and 2D band is relative to the vibration mode of the two photonic lattices rather than the defect); XRD patterns of C/Ni-10 and C-10 (**c**), and (**d**) G/Ni-10.

As shown in Figure 2a,b, the Raman spectrum of both C/Ni-10 and G/Ni-10 exhibits typical D and G band of carbon materials, confirming that carbonaceous coatings were deposited on bulk Ni substrate via different deposition techniques. However, distinguished from that of FDC, graphene coating displays strong peaks for the G band at 1581 cm^−1^ and the 2D band at 2706 cm^−1^, but a very weak peak for the D band at 1352 cm^−1^. Normally, the D band refers to the defect or disorder structure, whereas G band originates from the in-vibrations of sp^2^-bonded carbon atoms [32,33]. The 2D band represents the vibration mode of the two photonic lattices rather than the defect [33]. The intensity ratio (I_2D_/I_G_) of the graphene coating is 0.45, which is much less than 1, implying its multi-layered structure. As calculated by the spectrum in Figure 2a,b, the intensity ratio (I_D_/I_G_) of the FDC and graphene coating is 1.3:1 and 0.14:1, respectively. These results indicate the presence of rich defects in the FDC layer, in contrast to the high regularity of the graphene coating fabricated via CVD with the same carbon source. In XRD patterns of C/Ni-10 and G/Ni-10 (Figure 2c), sharp peaks at 2θ = 44.5°, 51.8° and 76.4° correspond to (111), (200) and (220) plane of the polycrystalline Ni [18,22,34], respectively. Due to the strong Ni crystalline peak originating from the bulk Ni plate in C/Ni-10, and the amorphous state of the FDC layer, the peak of carbon is hardly defined. However, after the removal of the Ni substrate, a broad peak centered at 24.5° could be observed for C-10 (Figure 2d). It is related to the carbon (002) plane, confirming the formation of amorphous carbon on the bulk Ni plate. Besides the three strong peaks of polycrystalline Ni in the pattern of C/Ni-10, G/Ni-10 shows an additional sharp peak at 2θ = 26.6° (Figure 2c), which originates from the (002) plane of the deposited graphene film (Figure 2d), confirming its high crystalline structure. It is found that the intensities for all the three Ni peaks increase evidently after both the flame coating and CVD process, and G/Ni-10 exhibits the strongest peaks among them, indicating the re-crystallization of the bulk Ni substrate during the coating processes. Interestingly, the intensity ratio of Ni (200) peak to Ni (220) peak increases dramatically from 3.1 for bare Ni to 4.2 for G/Ni-10, whereas the ratio for C/Ni-10 (3.0) remains almost the same as that of bare Ni plate (Figure 2c). Obviously, due to the low carbon-growing temperature required for our flame method, the influence of the flame coating process on the crystalline structure of the bulk Ni substrate is insignificant. This could be one of the advantages of the flame coating method, which suggests its promising application in some specific surface engineering. For instance, the mechanical properties of the carbon fibers changed little after the flame growing CNTs onto them, which benefits their application in the carbon fiber-reinforced polymer composite laminates [35].

**Figure 3:**
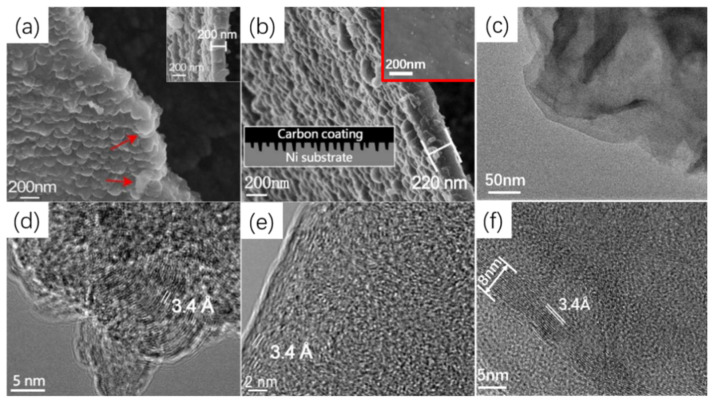
SEM images of the inner surface of (**a**) C-5, (**b**) C-10, insets in (**b**) are the sketch illustrated interlocked interface and the outer surface of C-10, respectively; TEM images of C-10 (**d**,**e**); TEM images of G-10 (**c**,**f**).

In order to explore the physical structure of carbon coating more comprehensively, FDC layers were prepared by the removal of Ni substrate from the flame coated samples. The remainder is an integral free-standing thin film. We found that its thickness increased rapidly with the flame time in the first 5 min, and then increased very slowly. According to the SEM analysis (Figure 3a,b), the thickness of the FDC layer was about 90, 150, 200 and 220 nm for C/Ni-1, C/Ni-3, C/Ni-5 and C/Ni-10, respectively. Interestingly, it is found that the two sides of the FDC layer displayed different morphologies. As shown in Figure 3b, the inner side surface of the carbon layer facing the Ni plate is much rougher than that of the side facing the ethanol flame (inset in Figure 3b). Many nanosheets can be observed clearly on its inner side surface, and they seem to be aligned vertically to the metal substrate. This indicates the vertical growth of these carbon sheets during the flame coating process. The thickness of these sheets is in nano-scale, as highlighted by the red arrow in Figure 3a. This means that some carbon sheets are grown from inside of the Ni substrate, and the formed unique nano-interlocking C/Ni interface has been sketched as the inset in Figure 3b. This interlocked interface is expected to provide the coating not only with the superior adhesion to substrate but also the effective hindrance to the crack propagation in the coating, as well as to the diffusion of the corrosives along the interface. HRTEM images of the nanosheets in Figure 3d,e demonstrate that they are actually graphitic carbon sheets with the lattice spacing distance of 3.4 Å, corresponding to the graphite (002) facet.

In contrast to the FDC layer with a unique hierarchical surface structure revealed here, both sides of the graphene layers growing on Ni following CVD usually exhibit a smooth surface [36]. Figure 3c shows the TEM images of a folded graphene film of G-10. Its thickness is about 8 nm, which comprises of more than 20 layers, as shown in Figure 3f. It is much thinner than the FDC obtained with the same growing time. Evidently, the FDC grew much faster than that of graphene, although the flame deposition temperature (400–450 °C) was much lower than that for the CVD of graphene (1000 °C). Unlike the amorphous state of the FDC layer (Figure 2d and Figure 3d,e), the graphene sheet exhibits more continuous and ordered crystalline structure, with the lattice spacing distance around 3.4 Å (Figure 3c).

### 3.2. Scratching Resistance of Carbon Coating and Micro-Hardness of the Amorphous Carbon and Graphene-Coated Ni Plates

**Figure 4:**
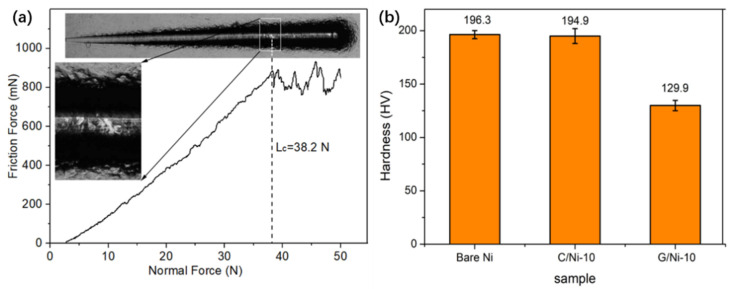
(**a**) Diagram of the scratch test curve of C/Ni-10 with inset optical images of the scratch track; (**b**) Hardness value of bare Ni, C/Ni-10 and G/Ni-10.

The scratch resistance of the coating was very important for its practical application, and was studied by a scratch test in this work. Figure 4a shows the scratch track as well as the in situ-recorded diagram of friction force versus normal force. As shown by the inset optical image of the magnified scratch track in Figure 4a, the metal substrate was exposed when the FDC layer slipped over on the substrate, while the detected friction force dropped suddenly. The critical load (Lc) corresponding to cohesion and adhesion strength between the coating and substrate was defined at this point where the failure occurred [37,38]. The Lc of the FDC coating was measured to be as large as 38.2 N, demonstrating its good anti-scratching performance and strong adhesion to the metal substrate. It is obviously larger than that of the conventional diamond-like carbon [2,3] and graphite-like carbon deposited by magnetron sputtering, but less than that of its counterpart incorporated with the CrC interlayer [5]. This indicates the major contribution of the coating/metal interface with a special nano-structure or the chemical covalent bonds to the scratch resistance of the coating.

Furthermore, the mechanical properties of the coated Ni substrates were measured to check the influence of the carbon deposition process on them, in consideration that either the FDC or CVD of graphene was carried out at high temperature. As shown in Figure 4b, the micro-hardness of FDC-coated Ni samples remains almost the same as that of bare Ni, and both of them were much higher than that of G/Ni-10, which was greatly reduced by about 30%. This phenomenon should be ascribed to the higher temperature for the CVD of graphene (1000 °C) than that of the flame coating method (<450 °C), which resulted into the different heat treatment of bare Ni plate substrate. As mentioned above, after the coating process, a greater change occurred in the crystalline structure for G/Ni-10, whereas little happened in the C/Ni-10 sample (Figure 2c). Although the exact mechanism remains unknown, these results were believed to coincide with the well-known finding that high-temperature heat treatment of metal samples usually results in metal re-crystallization, as well as the variation of their mechanical properties.

### 3.3. Corrosion Resistance of the Amorphous Carbon and Graphene-Coated Ni Plates

**Figure 5:**
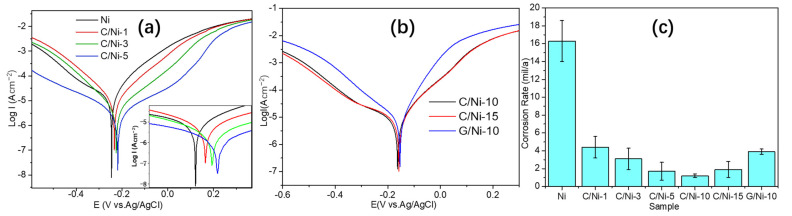
(**a**,**b**) Polarization curves of bare Ni and coated Ni samples, (**c**) the dependence of the corrosion rate of the samples on the coating type.

The corrosion inhibition performance of the carbon-based coating was characterized by potentiodynamic polarization tests. Figure 5a,b show Tafel plots of both bare Ni and coated Ni in 0.1 M HCl. As shown in Figure 5a, with the FDC layer, the polarization curves shifted in a positive direction along with the decreasing corrosion current density. Evidently, the i_corr_ decreased with the flame time up to 10 min (Figure 5a–c). The i_corr_ and corrosion rate (CR) are shown in Table 1, respectively. Obviously, the FDC growing for only 1 min in the ethanol flame could reduce CR to nearly a quarter of that of bare nickel plate. Moreover, the i_corr_ of C/Ni-10 was only 2.8 μA cm^−2^, which meant a roughly 13-fold improvement in the corrosion resistance over the bare Ni (38.3 μA cm^−2^). Additionally, when the flame time exceeded 5 min and the thickness of FDC layer was close to 200 nm, the effect of the flame time on its anti-corrosive performance became insignificant. For instance, either the i_corr_ or CR of C/Ni-5 was close to that of C/Ni-10 (Table 1). On the other hand, extending the time to 15 min deteriorated its corrosion inhibition performance as the CR of C/Ni-15 (1.9 mil a^−1^) was much larger than that for C/Ni-10 (1.2 mil a^−1^). This indicates that 10 min is the optimized time for the fabrication of the FDC layer with highest anticorrosion properties. Moreover, it is noted that the polarization curve shifted in a negative direction in comparison with those of both C/Ni-10 and C/Ni-15 (Figure 5b). And both i_corr_ and CR of the G/Ni-10 sample are also much larger than those of C/Ni-10 (Figure 5c and Table 1). This indicates that despite the same type of carbon source and growing time for both coating processes, the FDC coating of C/Ni-10 exhibited a higher anti-corrosion performance than that of its graphene counterpart. Corrosion protection efficiency (PE) has been utilized to normalize anti-corrosion performance of coatings and it is obtained by: PE = 100% × (i^0^_corr_ − i^c^_corr_)/i^0^_corr_, where i^0^_corr_ and i^c^_corr_ represents bare Ni and the coated Ni plates, respectively. Particularly, the PE of the FDC (92.7%) is much larger than that of G/Ni-10 (75.6%), and those values reported in the literature [11,15].

**Figure 6:**
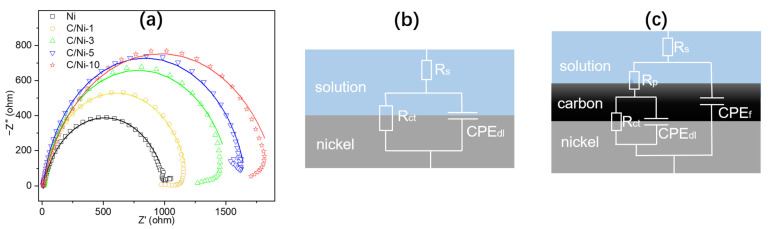
(**a**) EIS plots and fitted curves; Equivalent circuit of (**b**) bare Ni and (**c**) the coated Ni samples.

An EIS test and simulation analysis were performed to study the anti-corrosion behavior of the FDC coating. Scatter diagrams in Figure 6a present Nyquist plots of those samples with or without coating. Each diagram had been matched with a fitted curve which corresponds to an equivalent circuit, as shown in Figure 6b,c, respectively. All the fitted values of the elements in the equivalent circuits are displayed in Table 2. In general, the diameter of the semicircle is inversely proportional to the corrosion rate [39]. Therefore, the gradually increased diameters in the curves with the flame time in Figure 6a are highly coincident with the variation of CR values in Figure 5c and Table 1. The plot of bare Ni was simulated with a basic equivalent circuit consisting of two resistances and a constant phase element (Figure 6b). R_s_ represents the solution resistance. R_ct_ is defined as the electron transfer resistance at the metal/solution interface. Constant phase element (CPE) is an imperfect double-layer capacitance with a constant phase n (−90 × n°) formed by the dispersion effect. The value of n is related to the topography of the substrate surface [40,41]. An electrode with a smooth and homogeneous surface has a constant phase n = 1, which could be represented with a semicircle without depression. Additional elements R_p_ and CPE_f_ in the equivalent circuit of carbon-coated Ni samples represent the coating resistance and film capacitance, respectively (Figure 6c). Fitted values of R_p_ and R_ct_ can be used to evaluate the corrosion inhibition properties of the coating [42]. As shown in Table 2, both R_p_ and R_ct_ increase with the flame time. Evidently, the C/Ni-10 has the highest corrosion resistance, which coincides with the results of the potentiodynamic polarization test.

**Figure 7:**
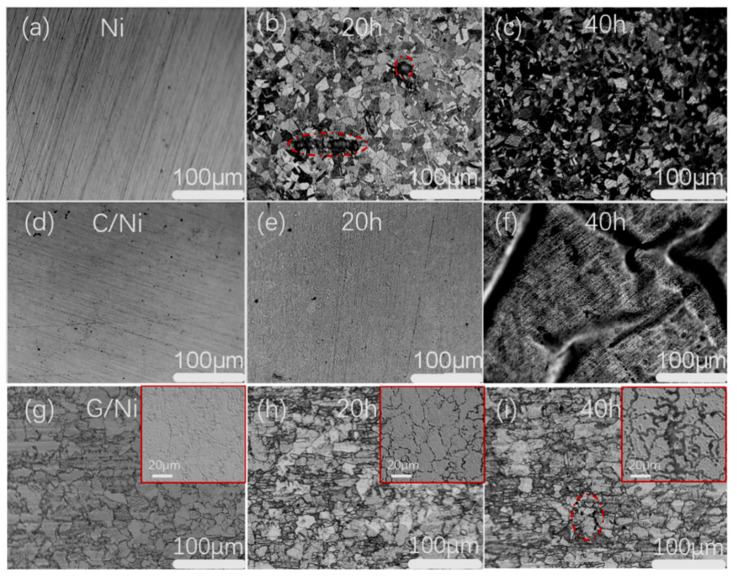
Optical images of the sample surface: bare Ni before (**a**) and after the immersion test for 20 h (**b**) and 40 h (**c**); C/Ni-10 before (**d**) and after the immersion test for 20 h (**e**) and 40 h (**f**); G/Ni-10 before (**g**) and after the immersion test for 20 h (**h**) and 40 h (**i**) together with their inset SEM images.

To explore the long-term anti-corrosion performance of the coated Ni samples, both bare Ni and coated Ni plates were immersed in 0.1 M HCl for a long period. The morphology evolution of metal surface with or without the coating during the immersion is presented in Figure 7. Evidently, the bare Ni plate was corroded, with the appearance of the distinctive inter-granular and localized corrosion after 20 h (Figure 7b). The Ni grain boundaries could be observed clearly, which meant that the metal surface was corroded preferentially along the grain boundaries. Some deep corrosion pits caused by the pitting corrosion could be found occasionally (see the highlighted circle area in Figure 7b). In contrast to the severely corroded bare Ni, the neat and smooth surface of C/Ni-10 was retained after the same immersion time (Figure 7e), confirming the significant protective performance of the FDC coating. Further increasing the corroding time to 40 h, the bare Ni was corroded more heavily (Figure 7c). On the contrary, the surface of C/Ni-10 changed little except for the appearance of some wrinkles (Figure 7d). This implies the occurrence of partial delamination of the coating from the metal substrate. The phenomenon is likely caused by the hydrogen evolution from the corrosion reaction between Ni and HCl, which took place at the coating/metal interface in a certain restricted area. Note that almost no breakage or failure in the FDC layer was observed (Figure 7d), implying the good integrity and mechanical flexibility of the FDC layer. For the graphene coating, the Ni grain boundaries of G/Ni-10 could be clearly observed with the optical microscope (Figure 7g), which could be due to the excellent optical transparency of the thin graphene coating. The irregular Ni grain size in the G/Ni-10 decreases greatly after 20 h, as shown in Figure 7h. With the immersion time being prolonged to 40 h, some cracks (highlighted by the red dotted line in Figure 7i) appear. In order to further reveal the corrosion process of the graphene-coated samples, SEM analysis was also utilized. Distinguished from the optical images, the surface morphology of the graphene coating could be clearly observed. As shown in the SEM images inset in Figure 7g–i, the grain boundaries of the G/Ni-10 sample become increasingly larger with longer immersion time. This confirms the grain boundary corrosion mechanism of the graphene coated sample. Based on both the optical and SEM images, it could be concluded that corroding starts from the Ni grain boundaries just under those of the graphene layer, and parts of the graphene will be delaminated from the substrate with the corrosion.

**Figure 8:**
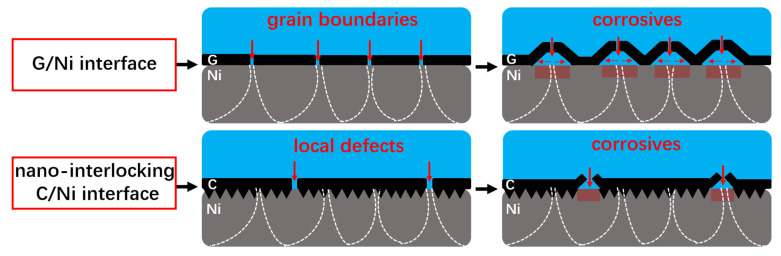
Schematic illustrating the corroding process occurred at the coating/metal substrate interface for the FDC and graphene-coated Ni samples.

Based on the experimental tests, the corresponding mechanisms of surface protection performance for both carbon-based coatings are proposed. For graphene coating with a plain interface and lots of grain boundaries, the corroding process starts from the grain boundaries (as shown in Figure 7h,i) and tends to spread throughout the coating/metal interface with long-term exposure to the corrosives. It is likely accompanied with the unrestricted delamination of the coating layer due to the plain coating/metal interface, as depicted in Figure 8. As a comparison, for the FDC-coated samples, the vertically aligned carbon sheets in the interface could restrict the diffusion of the corrosives. This will alleviate the crack propagation or the delamination of the FDC layer originated from the corrosion reaction, and thus only limited corrosion occurs under the same circumstances. Moreover, since the graphene coating has lots of grain boundaries, the graphene-coated samples have many weak points, and the entire surface starts to be corroded once the corrosives penetrate through these weak points (Figure 7h,i). In contrast, as demonstrated in Figure 7e,f, the FDC-coated samples start to be corroded only at limited local defective points, which are much fewer than the rich grain boundaries in their graphene counterparts. Therefore, FDC layers with this unique interlocking interface could endow them with superior anti-corrosive performance over that of graphene coating.

## 4. Conclusions

A simple and convenient flame coating method was developed to deposit the anti-corrosive carbon layers on bulk Ni substrate. The FDC layer displayed a unique 3D hierarchical structure, forming a nano-interlocking interface between the coating and metal substrate. For comparison, graphene coating fabricated via CVD with the same fuel and growing time was also prepared. Results indicated that the optimized coating time was 10 min, and the resulting carbon coating displayed a high protection efficiency of 92.7%, which was much larger than that of the graphene coating (75.6%) under the same test conditions. The scratch test result confirmed the excellent scratch resistance of the FDC layer and its good bonding to the substrate. The anti-corrosive mechanism of both the FDC and graphene coating were analyzed and explained. Additionally, due to the lower temperature required for growing FDC than that for graphene coating, the micro-hardness of the FDC-coated bulk Ni plate remained unchanged, in contrast to its graphene-coated counterparts (reduced by ~30%). The excellent anti-corrosion of the flame-treated metal substrate is believed to be due to the formation of the homogeneous FDC layer along the metal surface and the unique coating/substrate interlocking interface, in contrast to the evident grain boundary corrosion mechanism in the case of graphene coating.

## Figures and Tables

**Table 1:** The i_corr_, CR and PE of the samples.

Sample	i_corr_ (μA cm^−2^)	CR (mil a^−1^)	PE (%)
Bare Ni	38.3	16.3	-
C/Ni-1	10.3	4.4	73.1
C/Ni-3	7.3	3.1	80.9
C/Ni-5	4	1.7	89.6
C/Ni-10	2.8	1.2	92.7
C/Ni-15	4.5	1.9	88.3
G/Ni-10	9.3	3.9	75.6

**Table 2:** Values of fitted elements in equivalent circuits.

Sample	R_s_ (Ω cm^−2^)	CPEdl (S s^n1^ cm^−2^)	n_1_	R_p_ (Ω cm^−2^)	CPEf (S s^n2^ cm^−2^)	n_2_	R_ct_ (Ω cm^−2^)
Ni	13.1	6.87 × 10^−5^	0.8	-	-	-	1000
C/Ni-1	11.0	5.20 × 10^−5^	0.95	73.0	3.05 × 10^−5^	0.9	1110
C/Ni-3	13.3	8.24 × 10^−5^	0.91	100.7	4.11 × 10^−5^	0.89	1328
C/Ni-5	9.7	5.23 × 10^−5^	0.92	152.0	1.41 × 10^−5^	0.89	1401
C/Ni-10	10.3	1.11 × 10^−4^	0.8	210.5	5.70 × 10^−5^	0.8	1717

## Data Availability

Experimental data from this study are available upon request.
